# Pilot study of the influence of self-coding on empathy within an introductory motivational interviewing training

**DOI:** 10.1186/s12909-020-1956-5

**Published:** 2020-02-10

**Authors:** Trevor Simper, Jon Agley, Mallori DeSalle, Jennifer Todd, Tapati Dutta

**Affiliations:** 1 Small Changes Healthcare, Perth, Western Australia; 2 Prevention Insights, Department of Applied Health Science, School of Public Health–Bloomington, Indiana University, 501 N. Morton St., Suite 110, Bloomington, IN 47404 USA; 3 Department of Social Work, College of Health and Human Services, Indiana State University, Terre Haute, IN USA; 4 Health Sciences Department, Fort Lewis College, Durango, CO USA

**Keywords:** Empathy, Motivational interviewing, MI, Health professionals, Education

## Abstract

**Background:**

Motivational interviewing (MI) is a framework for addressing behavior change that is often used by healthcare professionals. Expression of empathy during MI is associated with positive client outcomes, while absence of empathy may produce iatrogenic effects. Although training in MI is linked to increased therapeutic empathy in learners, no research has investigated individual training components’ contribution to this increase. The objective of this study was to test whether a self-coding MI exercise using smartphones completed at hour 6 of an 8-h MI training was superior in engendering empathy to training as usual (watching an MI expert perform in a video clip for the same duration at the same point in the training).

**Methods:**

This was a pilot study at two sites using randomization and control groups with 1:1 allocation. Allocation was achieved via computerized assignment (site 1, United Kingdom) or facedown playing card distribution (site 2, United States). Participants were 58 students attending a university class at one of two universities, of which an 8-h segment was dedicated to a standardized MI training. Fifty-five students consented to participate and were randomized. The intervention was an MI self-coding exercise using smartphone recording and a standardized scoring sheet. Students were encouraged to reflect on areas of potential improvement based on their self-coding results. The main outcome measure was score on the Helpful Responses Questionnaire, a measure of therapeutic empathy, collected prior to and immediately following the 8-h training. Questionnaire coding was completed by 2 blinded external reviewers and assessed for interrater reliability, and students were assigned averaged empathy scores from 6 to 30. Analyses were conducted via repeated-measures ANOVA using the general linear model.

**Results:**

Fifty-five students were randomized, and 2 were subsequently excluded from analysis at site 2 due to incomplete questionnaires. The study itself was feasible, and overall therapeutic empathy increased significantly and substantially among students. However, the intervention was not superior to the control condition in this study.

**Conclusions:**

Replacing a single passive learning exercise with an active learning exercise in an MI training did not result in a substantive boost to therapeutic empathy. However, consistently with prior research, this study identified significant overall increases in empathy following introductory MI training. A much larger study examining the impact of selected exercises and approaches would likely be useful and informative.

## Background

### Motivational interviewing (MI)

Motivational Interviewing (MI) has a 35-year research history and is considered an efficacious clinical framework for resolving ambivalence and addressing behavior change, especially related to behavioral healthcare and addictions [1]. For example, MI is often included as an element in education and training on screening, brief intervention, and referral to treatment (SBIRT) [2]. As research on MI training and applications has progressed, increasing focus has been placed on the positive influence of therapeutic empathy on MI-consistent counseling behaviors [3], synchrony of language used between client and counselor [4], direct client-level behavioral outcomes [5], and general cohesion with the spirit of MI [6]. Notably, low therapist empathy may predict poor treatment outcomes [5]. There is therefore value in focusing specifically on acquisition of therapeutic empathy within MI training.

At the same time, measurement of MI training outcomes is complicated by the fact that training formats vary in terms of delivery and methods. For example, one meta-analysis of 28 MI training studies identified seven studies lasting fewer than 8 hours, 16 studies lasting between nine and 16 h, and five studies featuring extended timeframes [7]. MI trainings typically are delivered in a workshop format, though trainings can also include add-ons such as teleconferencing and booster sessions [8]. Research has indicated that a variety of workshop-driven formats, including those incorporating feedback and coaching, but also standalone workshops, produce superior proficiency to self-study controls [9]. MI skills development appears to be more sustainable when coaching and feedback are provided post-training [8]. Of particular interest for this study, researchers have also used the Helpful Responses Questionnaire (HRQ) [10], a measure of learner empathy, as a means of assessing the impact of MI training [11–13]. This work has generally found that MI training improves HRQ scores by a significant and meaningful amount.

### Teaching techniques within MI workshops

The existence of a formal Motivational Interviewing Network of Trainers (MINT) and competency requirements [14] provides some internal consistency of training workshop components. MI workshops with a MINT trainer often begin with a two-day workshop (e.g., [15]). The workshop generally includes didactic content, role-play and real-play (role-play in which the individual processes a scenario as him/herself in a realistic context), and video observation of expert MI practitioners. Role-play and real-play are thought to be especially important, not only in terms of practicing applicable skills, but also because the type of learning that occurs in the context of self-reflection produces stronger outcomes than those attributed to an exclusively didactic style of delivery [16].

### Purpose

The present investigation began with a supposition based on observations of the lead author that a self-coding exercise was the point in his own MI training workshops where learners seemed to grasp the clinical application of MI. There has been little research into MI self-coding within workshops, with 1 notable exception [17], and no research has been conducted regarding the effects of specific components of MI training workshops on development of learning outcomes, including therapeutic empathy. At the same time, the importance of investigating ‘within workshop’ MI training elements was noted in a recent editorial outlining necessary directions for MI research [18]. General health and medical education research suggests that a self-coding exercise following a brief real-play may be an especially effective MI training element, as it combines aspects of experiential adult learning [19, 20] and structured assessment following role-play [21]. However, there is no extant research regarding the effect on learner outcomes, including development of therapeutic empathy, attributable to any single component of an MI workshop.

This paper therefore describes a pilot study conducted among undergraduate students in both the United States (USA) and United Kingdom (UK). The study investigated whether a standard eight-hour MI workshop with an MI self-coding exercise (intervention) delivered 6 hours into the workshop was superior in building participant empathy when compared with the same workshop with students watching a video of an MI expert performing MI (control) in place of the self-coding exercise.

## Methods

### Ethics

The institutional review boards at both study sites approved this study (Sheffield Hallam University, #ER5231303, and Indiana State University, #1151112–2).

### Participants

During the semester designated for the study, all students who either registered for and attended an undergraduate screening, brief intervention, and referral to treatment elective class within the Department of Social Work (of which 8 h were MI training) at Indiana State University, USA, or who registered for and attended a third year undergraduate nutrition class (of which 8 h were MI training) at Sheffield Hallam University, UK, were recruited. These potential participants were healthcare students either studying to become social workers or nutritionists. The MI approach can be used by a wide variety of fields, and has been taught to numerous healthcare disciplines, including social work and nutrition [22]. Thus, the only exclusion criterion was refusal to participate after reading the study information sheet. Excluded students still participated in the eight-hour training but were not asked to complete any study questionnaires.

### Interventions

All participants first received a six-hour training block of introductory MI training conducted by one of two study authors (TS and MD), who are members of MINT; the training content was commensurate with recommendations by MINT for an introductory MI training [23]. Then, participants randomized to the intervention were led to a separate area to complete a self-coding exercise with a partner. Participants randomized to the control group remained in the classroom and watched a video of an expert performing MI. All participants completed the remainder of the MI training (approximately 100 additional minutes) after completing either the intervention or the control exercise.

The self-coding intervention was a real-play experience where each participant was asked to identify an aspect of their lives that they felt ambivalent about changing and were comfortable both discussing with a classmate and recording. Exemplar topics included physical activity, diet, smoking, or alcohol consumption, but no topic was specifically excluded. Each member of each pair counseled the other about the identified behavior using applicable MI skills. Participants were instructed to audio record their session as the helping professional. Audio recording was completed using each participant’s personal smartphone (using memo recording, voice recording, or a camera function without video enabled), with recording devices placed between members of the pair. After recording was completed for both partners, each participant listened to his/her own recording (where they were the helping professional) and completed a self-coding exercise using a coding sheet developed by the first author (see Additional file 1).

For the coding exercise, participants were instructed to mark the appropriate box for both MI-consistent (e.g., Affirmations) and MI-inconsistent (e.g., Authoritarian statements) behaviors using tally marks to indicate the number of times each behavior occurred. Space was also provided for participants to add examples. Participants were told that they could pause, rewind, and re-play the recording as needed. Finally, participants were asked to reflect to themselves, after completing the coding sheet, what went well during their recorded sessions and what, if anything, they would change about their practice in subsequent sessions. To reduce social desirability bias, the self-coding sheet was neither collected nor evaluated by the instructor.

### Study structure

This study was a pilot project using a two-group parallel, randomized controlled design with 1:1 allocation.

### Outcome measure

The HRQ is a six-item free-response questionnaire measuring therapeutic empathy [10] and commonly used to assess learner outcomes in MI training [7]. Participants completed the HRQ at the beginning of the study, and again at the end of the eight-hour training. The tool asked participants to respond to a series of vignettes in an open-ended style, and they were instructed to “think about each paragraph as if you were really in the situation… in each case write the *next thing* that you would say if you wanted to be helpful” (p. 444) [10]. HRQ scoring was completed by independent expert reviewers using standard criteria; each open-ended response was scored by external reviewers from one to five, with a ‘1’ not only indicating no reflection, but also a ‘roadblock’ (a response that interrupts dialogue between counselor and client), and a ‘5’ indicating a complex reflection of the client’s feeling (or similar metaphor) with no roadblock content present. Total scores therefore can range from 6 to 30. The reviewers were not part of the study team and were blinded to both the group assignment (intervention/control) and the administration time (pre/post). HRQ scores were the mean of coders’ ratings for each individual at each administration point.

### Interrater reliability

Interrater reliability of the two coders was calculated at baseline and follow-up using Krippendorff’s alpha [24] with the level of measurement set as interval and 1000 bootstrap samples used to generate confidence intervals. This metric can range from zero to one, with ‘1’ representing perfect reliability. At both baseline and follow-up, coders exhibited excellent agreement (Baseline: α = .965, LL_95%CI_ = .944, UL_95%CI_ = .983; Follow-Up: α = .961, LL_95%CI_ = .940, UL_95%CI_ = .975).

### Sample size and randomization

There was no precedent for an estimated effect size of a training modification such as this intervention on learners’ therapeutic empathy. Because of this, and given the naturalistic setting of our pilot study within preexisting university classes, the protocol did not utilize an a priori power analysis, choosing instead to invite all enrolled students to participate in the study (*n* = 79 eligible students, *n* = 53 analytic sample; see *Participant Flow*).

In the US cohort, simple randomization was achieved using facedown playing cards, and in the UK it was achieved using a computerized random number generator to separate participants [25]. We selected which card suits (US) or numbers (UK) were intervention and control indicators prior to using the mechanisms to sort participants. In the US, an assistant, rather than a member of the study team, passed out the facedown cards. In the UK, a study team member applied the randomly sequenced numbers to the participants as generated. In this way, allocation concealment can be inferred. All individuals generating outcome measure scores (the ‘coders’) were blinded to both group assignment and measurement point (pre/post).

### Statistical assumptions and methodology

The outcome of interest was the interaction effect of HRQ administration time and group allocation, as it was expected that both groups would naturally display improved therapeutic empathy, but that the experimental group’s improvement would be significantly greater. Thus, repeated measures ANOVA was used to generate statistical estimates of effect size and significance via the general linear model, IBM SPSS Statistics 25, and then the plot of means was interpreted [26, 27]. Separate analyses of pre-post data by group were completed using Student’s t-test and included in Table 1 to more clearly illustrate changes in measured therapeutic empathy over time as a result of the full training, but these analyses should not be used to interpret the effects of the intervention.

**Table 1:** Comparison of pre and post-training scores by group assignment

	Pre Mean (SD)	Post Mean (SD)	Post-Pre Mean Difference (95% CI)	Effect Size (Cohen’s D; 95% CI)	Significance (*p*-value)
Control (*n* = 26)	7.00 (2.74)	12.48 (4.40)	5.48 (3.77–7.19)	1.50 (0.63–2.37)	<.001
Experimental (*n* = 27)	8.17 (3.79)	15.41 (4.05)	7.24 (5.44–9.04)	1.85 (0.95–2.75)	<.001

Data exhibited high levels of skewness and kurtosis, especially at baseline (skew = 2.346 [SE = .327]; kurt = 4.549 [SE = .644]), and Shapiro-Wilk tests of normality indicated violations in both cases (Baseline w = .544, df = 53, *p* < .001; Follow-Up w = .928, df = 53, *p* = .003). This is typical for pilot data of this type [28]. There was one univariate outlier slightly exceeding an absolute value of Z = 3.29, but this case did not meaningfully affect overall skewness and kurtosis, so it was retained [29]. Multiple transformations (log, modified log, reciprocal, exponential) were attempted but were unable to achieve non-significant Shapiro-Wilk test values. However, parametric comparison of means is generally robust to violations of normality in the absence of extreme outliers and at least 20 degrees of freedom [29]. Parametric tests also allow for estimation of effect size, in keeping with CONSORT 2010 recommendations [30]. Therefore, the planned comparison strategy was retained over the potential alternative of using non-parametric tests [31].

## Results

### Participant flow

**Fig. 1:**
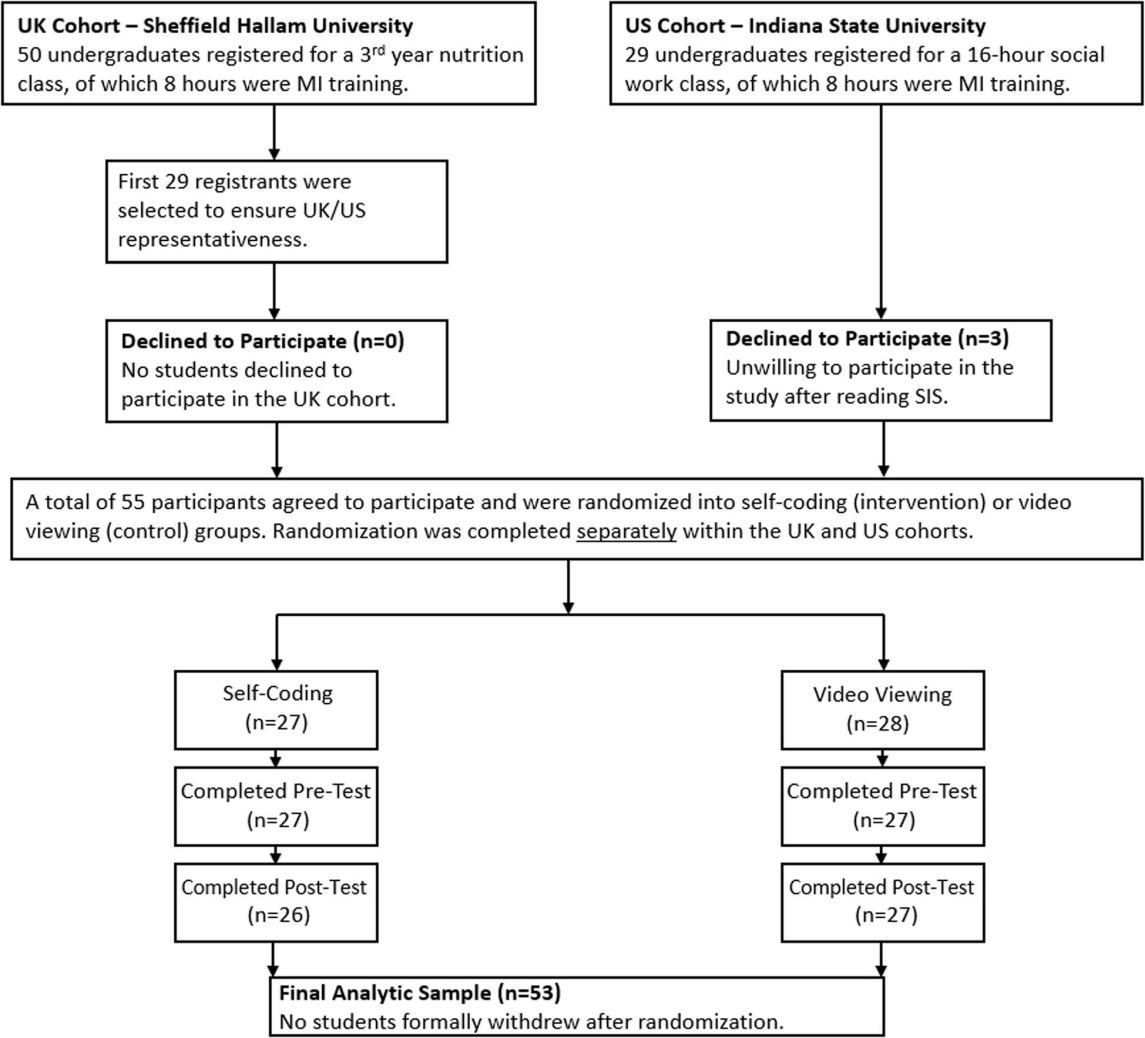
Participant Flow Chart

Seventy-nine undergraduates (*n* = 50 UK, *n* = 29 US) were eligible for this trial. Only the first 29 students in the UK arm were utilized for analysis to avoid potential overrepresentation bias from different instructors, field of study, or course location in the UK versus the US. After potential participants were provided with a study information sheet, three US students declined to participate. The remaining 55 students were randomized into the self-coding (*n* = 27) intervention group and the video viewing (*n* = 28) control group. One US student failed to complete the pre-test (but completed the post-test), and a separate US student failed to complete the post-test (but completed the pre-test). Both students were excluded from primary analyses but their data were included in calculations of interrater reliability. A full participant flow diagram is included as Fig. 1.

### Empathy characteristics

At baseline, both the control and experimental groups demonstrated little therapeutic empathy, with mean scores of 7.00 (SD = 2.74) and 8.17 (SD = 3.79), respectively, (within a possible range of 6 to 30). Both groups presented significantly improved empathy (*p* < .001) by the end of the MI training, with mean scores of 12.48 (SD = 4.40) and 15.41 (SD = 4.05), respectively (see Table 1).

### Primary analysis

A mixed ANOVA using the general linear model found a significant main effect for the MI training program across all students (F_1,51_ = 110.83, *p* < .001). The partial ƞ^2^ statistic (.685, LL_90%CI_ = .554, UL_90%CI_ = .757) suggested that the training resulted in a large increase in mean therapeutic empathy for all students, in aggregate. Although baseline differences between the control and experimental groups were, by definition, random, the between subjects main effect of group allocation was significant (F_1,51_ = 5.79, *p* = .020) with a partial ƞ^2^ statistic of .102 (LL_90%CI_ = .001, UL_90%CI_ = .240).

**Fig. 2:**
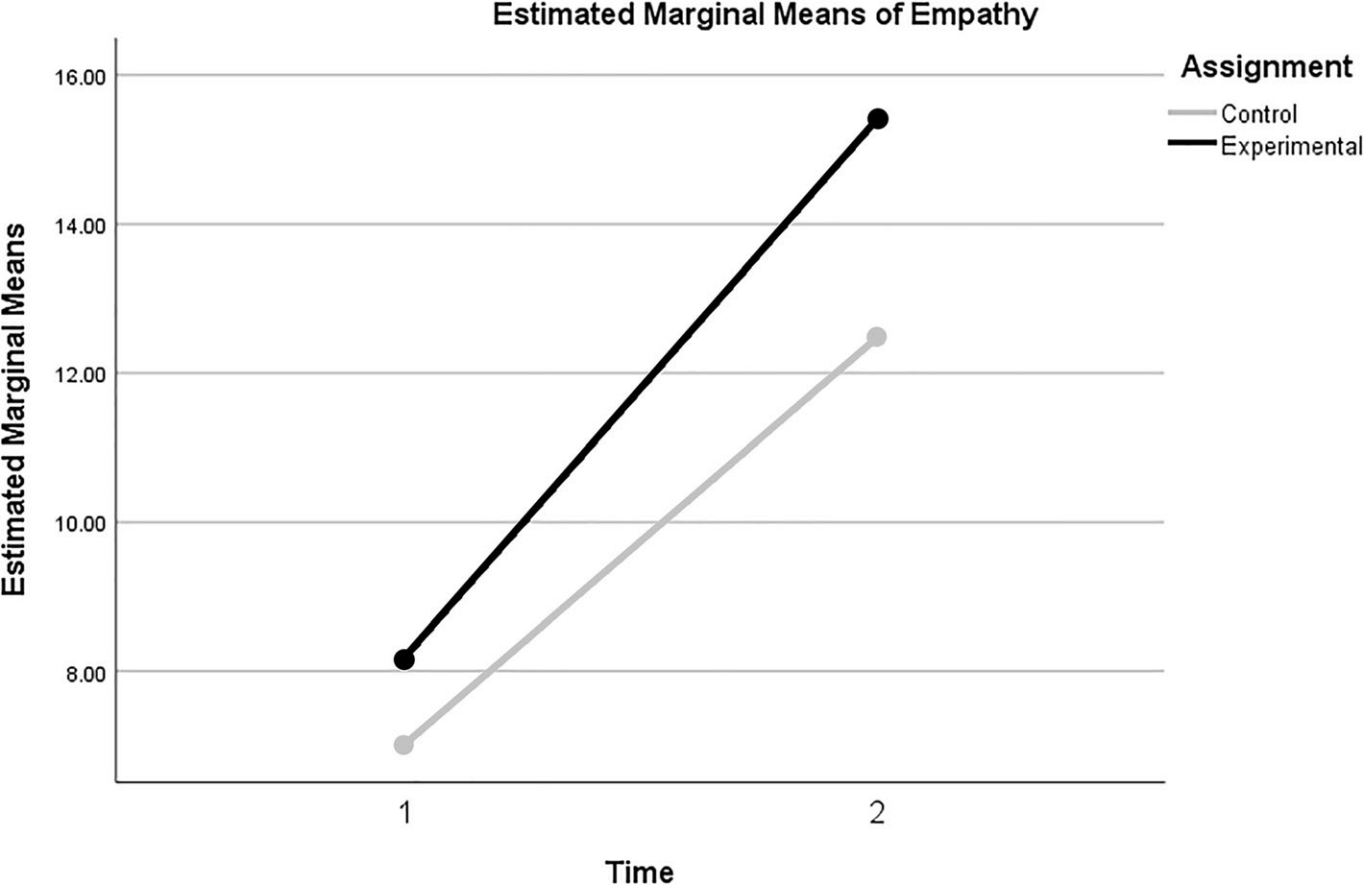
Graph of Estimated Marginal Means

The interaction effect measured the degree to which the change in therapeutic empathy over time was different for the experimental and control groups. This effect was non-significant (F_1,51_ = 2.12, *p* = .151), with a partial ƞ^2^ statistic of .040 (LL_90%CI_ = .000, UL_90%CI_ = .154), a small effect but one with potential practical implication [32] (see Table 2). The plot of estimated marginal means (Fig. 2) illustrates the implications of the GLM output, as the slope of the experimental group’s increase is somewhat sharper, but both groups increased relatively uniformly.

**Table 2:** Mixed ANOVA (General Linear Model)

	Pre Mean (SEM)	Post Mean (SEM)	Post-Pre Mean Difference (95% CI)	Test Value (F)	Effect Size (Partial ƞ^2^)	Significance (*p*-value)
Combined Sample (*n* = 53)	7.58 (.47)	13.94 (.58)	6.36 (5.15–7.57)			
Within Subjects (Time)				110.83	0.69	<.001
Interaction (Time x Group)				2.12	0.04	.151
Between Subjects (Group)				5.79	0.10	.020

## Discussion

### Interpretation

The notion that experiential learning is useful alongside or instead of didactic delivery of information is not a new concept. Role-playing and self-evaluation are often used when developing adult learning curricula [33]. The question of whether a single exercise within a MI workshop might, by itself, increase therapeutic empathy above more passive information transfer via observation of an expert, was heretofore unexplored. This pilot study used randomization and a control group to test the hypothesis that a self-coding exercise at hour six of an eight-hour MI training was superior in building therapeutic empathy to watching a video of an MI expert performing MI. The study outcome did not support rejecting the null hypothesis.

While we had speculated that the isolated self-coding exercise might, in and of itself, result in a substantial boost in therapeutic empathy relative to passive learning, our measured effect was non-significant and small (.040), even at the upper bound of the 90% CI. One possible implication of failing to reject the null hypothesis may be that there is no one single point where learners experience a large increase in ability to express empathy, but rather that each separate component of the MI training synergistically builds on the others in increments, resulting in the aggregate gain in therapeutic empathy at workshop conclusion observed in this and other studies. An assessment of whether that is the case would require a larger sample size and, ideally, multiple study arms testing additional learning conditions and approaches.

In addition to the general finding about MI workshops, there are two supplemental areas where education research might be influenced. First, prior to this study, the range of realistic effects on therapeutic empathy that might be expected from a single exercise within an MI workshop was unknown. While it is not recommended to base study power analyses solely on effect sizes from pilot tests [34], data from this study suggest that a medium or large effect would likely not be reasonable to expect from a single training modification of this type. Second, our failure to reject the null hypothesis does not imply that the self-coding exercise did not support building therapeutic empathy, but rather that it was not measurably superior, within the context of an introductory MI training, to a passive learning exercise (video viewing). Madson and colleagues [18] described a need to: “seek to better understand the effective training ingredients.” For practitioners interested in this work, the present study is one of the first steps in this undoubtedly long and complex process.

### Strengths and limitations

This study has several limitations. First, outcomes were observed only among undergraduate students enrolled in universities, so extrapolation of the findings to other commonly-trained groups (e.g., experienced therapists) should be done with caution. Second, both the trainers involved in the present investigation are members of MINT, limiting generalizability to workshops run by trainers who are not MINT members (e.g. potentially less experienced). Third, prior experience with MI was not elicited at enrollment for this study. At the same time, since these were undergraduate courses, it is somewhat unlikely that any student would have had extensive prior MI experience. Finally, the study’s focus was solely on therapeutic empathy, so findings cannot be generalized to other potential outcomes from MI training, such as lower-level skills (e.g., use of affirmations). This study also has several strengths: The study included students from two different countries (USA and UK), and included students studying several different disciplines, allowing increased generalizability outside of the field of social work to other health-supportive fields that may use MI. We also note a correspondence with prior research on MI workshops that captured HRQ data, as the overall significance and effect size of the MI training on therapeutic empathy in this study mirrors that work [11–13]. This supports the overall validity of the study.

## Conclusions

Our findings suggest that a single active learning exercise within an MI workshop for undergraduate learners in social work and nutrition may not be superior to a passive learning exercise in building therapeutic empathy. However, the pilot study itself was eminently feasible, with few barriers to completion, even across continents, raising the potential of developing a larger and more thorough assessment of MI workshop content in order to optimize within-training outcomes across desired domains like empathy. Further, our findings continue to reinforce the probability that even brief (8-h) MI training workshops are likely to increase participants’ empathy.

## Supplementary information

**Additional file 1.** Motivational Interviewing Coding Sheet for Practice Sessions

## Data Availability

Data are available from the corresponding author on request.

## Abbreviations

HRQ: Helpful Responses Questionnaire
MI: Motivational Interviewing
MINT: Motivational Interviewing Network of Trainers
SBIRT: Screening, brief intervention, and referral to treatment
UK: United Kingdom
USA: United States of America
