# Supplementary material for: Pilot study of the influence of self-coding on empathy within an introductory motivational interviewing training
Source: BMC Med Educ. 2020 Feb 10;20:43. doi: 10.1186/s12909-020-1956-5 (PMC7011448; doi:10.1186/s12909-020-1956-5)
Supplement: Supplementary file 1 — Additional file 1. Motivational Interviewing Coding Sheet for Practice Sessions [file 12909_2020_1956_MOESM1_ESM.pdf]

Motivational Interviewing Coding Sheet for Practice Sessions

Interviewer____

Client ____

Observer/Coder(s)_____________________________________

**MI Consistent**<br><br>Write examples and count the number given during conversation.	Open Qs		Affirmations	Reflections	Summaries
**MI Inconsistent**	Closed ?s	Judgmental	Confrontational	Authoritarian	Lectures/ gives unsolicited advice

Sum Open Questions____________ **Sum Affirmations**_______________
Sum Closed Questions___________ **Sum Reflections**_________________
Sum Summarizations_________

TARGETS:
80 % of questions asked should be open questions.
Aim for 4 open questions to every closed question.
Aim for 2-3 reflections for every question regardless of whether it is closed or open.

COMMENTS:
